# Calcium and vitamin D supplementation and/or periodontal therapy in the treatment of periodontitis among Brazilian pregnant women: protocol of a feasibility randomised controlled trial (the IMPROVE trial)

**DOI:** 10.1186/s40814-019-0417-6

**Published:** 2019-03-05

**Authors:** Paula Guedes Cocate, Gilberto Kac, Berit L. Heitmann, Paulo Nadanovsky, Maria Cláudia da Veiga Soares Carvalho, Camila Benaim, Michael Maia Schlüssel, Maria Beatriz Trindade de Castro, Nadya Helena Alves-Santos, Amanda Farnum Baptista, Michael F. Holick, Rana R. Mokhtar, Alessandra Raymundo Bomfim, Amanda Rodrigues Amorim Adegboye

**Affiliations:** 1 Department of Bioscience and Physical Activity, School of Physical Education and Sports, Rio de Janeiro Federal University, Rio de Janeiro, Brazil; 2 Nutritional Epidemiology Observatory, Department of Social and Applied Nutrition, Institute of Nutrition Josué de Castro, Federal University of Rio de Janeiro, Rio de Janeiro, Brazil; 3 Research Unit for Dietary Studies at the Parker Institute, Bispebjerg and Frederiksberg Hospital, The Capital Region, Denmark and Section for General Medicine, Institute of Public Health, Copenhagen, Denmark; 4 Institute of Social Medicine, State University of Rio de Janeiro, Rio de Janeiro, Brazil; 5 Department of Social and Applied Nutrition, Institute of Nutrition Josué de Castro, Rio de Janeiro Federal University, Rio de Janeiro, Brazil; 6 Centre for Statistics in Medicine, Nuffield Department of Orthopaedics, Rheumatology and Musculoskeletal Sciences, University of Oxford, Oxford, UK; 7 Section of Endocrinology, Diabetes & Nutrition, Department of Medicine, Boston University School of Medicine (BUSM), Boston, MA USA; 8 Boston University School of Medicine (BUSM), Boston, MA USA; 9 Department of Psychology, Social Work & Counselling, Faculty of Education and Health, University of Greenwich, Old Royal Naval College, Park Row, London, SE10 9LS UK

**Keywords:** Calcium, Feasibility randomised controlled trial, Milk, Pregnant women, Periodontal therapy, Vitamin D

## Abstract

**Background:**

Periodontitis is a common oral inflammation, which is a risk factor for adverse pregnancy outcomes. Intakes of vitamin D and calcium are inversely associated with occurrence and progression of periodontitis. This study aims to assess the feasibility of a multi-component intervention, including provision of milk powder supplemented with calcium and vitamin D and periodontal therapy (PT), for improving maternal periodontal health and metabolic and inflammatory profiles of low-income Brazilian pregnant women with periodontitis.

**Methods:**

The IMPROVE trial is a feasibility randomised controlled trial (RCT) with a 2 × 2 factorial design with a parallel process evaluation. Pregnant women with periodontitis, aged 18–40 years and with < 20 gestational weeks (*n* = 120) were recruited and randomly allocated into four groups: (1) fortified sachet (vitamin D and calcium) and powdered milk plus PT during pregnancy, (2) placebo sachet and powdered milk plus PT during pregnancy, (3) fortified sachet (vitamin D and calcium) and powdered milk plus PT after delivery and (4) placebo sachet and powdered milk plus PT after delivery. Dentists and participants are blinded to fortification. Acceptability of study design, recruitment strategy, random allocation, data collection procedures, recruitment rate, adherence and attrition rate will be evaluated. Data on serum levels of vitamin D, calcium and inflammatory biomarkers; clinical periodontal measurements; anthropometric measurements; and socio-demographic questionnaires are collected at baseline, third trimester and 6–8 weeks postpartum. Qualitative data are collected using focus group, for analysis of favourable factors and barriers related to study adherence.

**Discussion:**

Oral health and mineral/vitamin supplementation are much overlooked in the public prenatal assistance in Brazil and of scarcity of clinical trials addressing these issues in low and middle-income countries,. To fill this gap the present study was designed to assess the feasibility of a RCT on acceptability of a multi-component intervention combining conventional periodontal treatment and consumption of milk fortified with calcium-vitamin D for improving periodontal conditions and maternal metabolic and inflammation status, among Brazilian low-income pregnant women with periodontitis. Thus, we hope that this relatively low-cost and safe multicomponent intervention can help reduce inflammation, improve maternal periodontal health and metabolic profile and consequently prevent negative gestational outcomes.

**Trial registration:**

NCT, NCT03148483. Registered on May 11, 2017.

## Strengths and limitations of this study

- To the best of our knowledge, this is the first feasibility trial to explore the acceptability of a multi-component intervention combining milk powder supplemented with calcium and vitamin D and periodontal therapy in Brazilian pregnant women.
- This study will contribute to the limited literature on the impact of milk intake, supplementation of calcium and vitamin D and periodontal therapy for improving maternal metabolic and inflammation status and periodontal health.
- The evaluation process includes qualitative data to support the assessment of barriers and facilitators related to the study feasibility.
- Follow-up for this study is limited to 6–8 weeks after birth due to study budget and timeframe. Ideally, longer-term follow-up would be preferable to allow for the initial effect of the intervention to be assessed in both mothers and infants.

## Background

Periodontitis is an inflammatory disease defined as a bacterial gum infection causing a breakdown of soft tissue and loss of tooth-supporting bone [1]. If untreated, periodontitis can cause tooth loss potentially resulting in low self-esteem, impaired speech and chewing and reduced ability to eat healthy and nutritious food [2]. Periodontitis is one of the most common oral conditions and is prevalent in both developed and developing countries reaching approximately 20–50% of the global population [3]. Pregnant women, due to hormonal changes, are prone to develop periodontitis or existing periodontitis may worsen with the progression of pregnancy [4]. In Brazil, the prevalence of moderate periodontitis among pregnant women has been estimated to be between 11% and 47% [5–8].

The inflammatory burden induced from periodontitis may have repercussions beyond the oral cavity, leading to low-grade systemic inflammatory status and metabolic disturbances, which can affect the course of gestation [9]. Increased levels of pro-inflammatory mediators such as interleukin-6 (IL-6), C-reactive protein (CRP) and matrix metalloproteinase 9 (MMP-9) have been observed among people with periodontitis [10–12].

Systematic reviews have shown that women with periodontitis are at higher risk of delivering preterm (< 37 weeks gestation) and low-birth-weight (< 2.5 kg) babies, [13–15] both of which are among the leading causes of neonatal deaths [16]. The biological mechanism underlying the link between periodontitis and adverse pregnancy outcomes is not fully known [17]. Periodontitis may cause bacterial translocation through blood circulation or production of inflammatory mediators associated with premature rupture of membranes and onset of delivery [18]. Additionally, it may initiate and propagate a framework for insulin resistance and ultimately the manifestation of gestational diabetes [9]. The vertical transmission of oral pathogenesis is also a concern, as children of mothers with periodontitis are at higher risk of acquiring periodontal pathogens [19].

Randomised controlled trials (RCT) investigating whether non-surgical periodontal therapy (PT) during pregnancy has the potential of reducing miscarriage, preterm birth and low-birth-weight incidence have shown conflicting results [20–23]. However, the majority of the RCTs have found no significant effect [20, 22, 23], probably due to the PT not being intense or early enough to prevent disease progression, suggesting that ongoing periodontal maintenance treatment throughout gestation might be needed [24].

The active form of vitamin D has an important action in relation to regulating calcium and skeletal homeostasis and also acts as an anti-inflammatory and anti-microbial agent that may be beneficial to periodontal health by antibiotic effects on periodontopathogens and inhibition of inflammatory mediators that contribute to the periodontal destruction [25]. Besides vitamin D, calcium has also been associated with lower occurrence of periodontitis, probably by prevention of alveolar bone loss and better natural dentition, i.e. the higher calcium consumption is associated with lower loss of tooth [26].

Calcium and vitamin D co-supplementation have been hypothesised to act in synergy rather than independently [27], since 1,25-dihydroxyvitamin D acts on the small intestine and kidneys to increase absorption of calcium [25]. Previous studies in an adult Danish population have shown that intakes of calcium within recommendations (1000 and 1200 mg/day for men aged 51–70 and > 70 years, respectively, and 1200 mg/d for women aged ≥ 51 years) are associated with lower risk of periodontitis and tooth loss only among those with higher intake of vitamin D (> 6.8 μg/d) [28, 29]. These studies suggest that calcium obtained from dairy sources was associated with benefits for periodontal health. In addition, a non-randomised clinical trial performed among Indian adults found that the group taking vitamin D (250 IU/day) and calcium (500 mg/day) supplementation for 3 months developed better periodontal health (higher bone density and lower gingival bleeding index) than the group that did not take supplementation [30]. However, larger studies are needed to evaluate the effectiveness of this strategy in pregnant women.

In Brazil, the prevalence of vitamin D insufficiency (defined as 25(OH) D levels < 20 ng/mL) in pregnant women ranges from 16.1 to 43% [31, 32]. Moreover, mean calcium consumption among adult Brazilian women is only 476 mg/d [33], which is below the dietary recommendations of 1000 mg/d [34].

Considering the high prevalence of periodontitis and vitamin D insufficiency and low calcium consumption among Brazilian pregnant women, the purpose of this study is to assess the feasibility and acceptability of a multi-component intervention, including provision of milk powder supplemented with calcium and vitamin D and periodontal therapy (PT), for improving maternal periodontal health and metabolic and inflammatory profiles.

### Objectives

The primary and secondary objectives are described in Table 1.

**Table 1:** Objectives of the IMPROVE feasibility trial

Primary	Secondary
1. Feasibility of recruitment strategy for local health service and participants	1. Estimates of effect size and variability to enable accurate sample size and power calculations for a definitive RCT
2. Time necessary to recruit participants	2. Identification of any differences across age groups and other socio-demographic factors with regards to attrition rate
3. Acceptability of the study design and random allocation	3. Identification of key barriers and enablers to adoption, and large-scale implementation, of the IMPROVE trial are
4. What the most appropriate inclusion/exclusion criteria are	4. Definition of required for economic analysis and if they can be collected reliably
5. Estimates of compliance, satisfaction, follow- up and attrition rates	5. The capacity of the research team to embed this study into routine practice in the Public Health Centres
	6. To determine the initial effects of the intervention, time and intervention-time interaction on inflammatory biomarkers concentrations and periodontitis status

## Methods/design

### Study design and setting

**Fig. 1:**
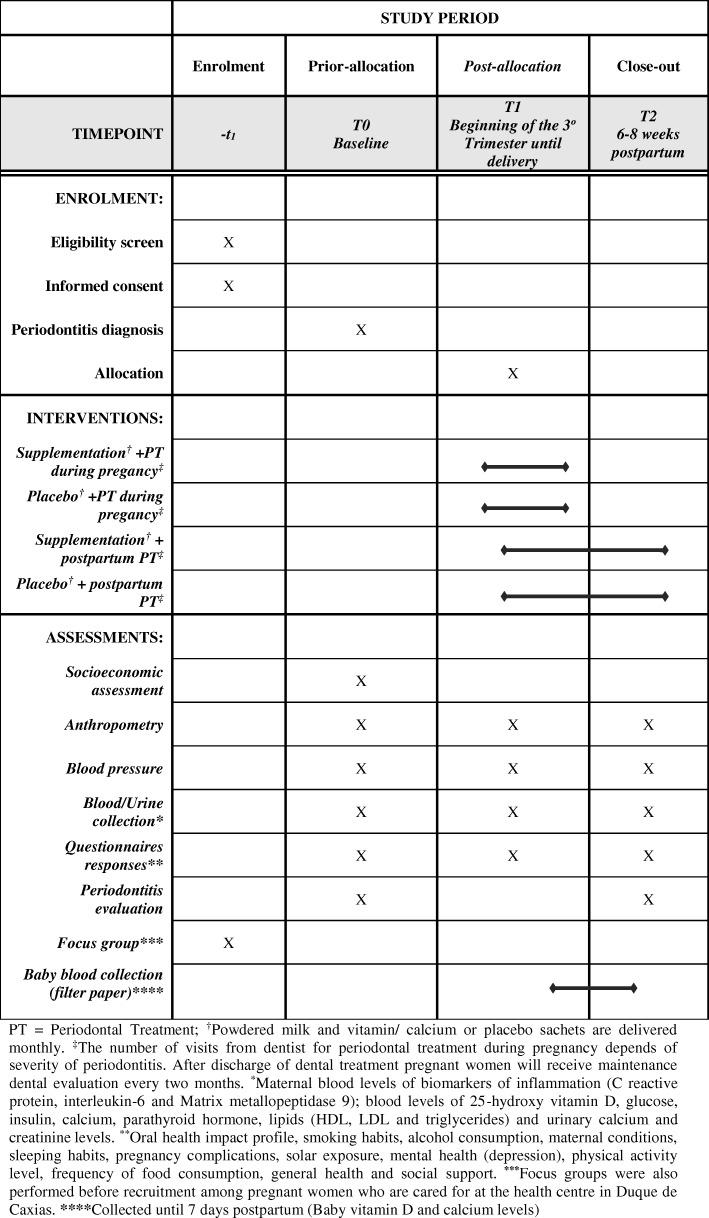
SPIRIT flow diagram of the IMPROVE feasibility trial

The IMPROVE trial is a 2 × 2 factorial feasibility RCT with a parallel process evaluation, i.e. the groups are not cross-over [35], conducted among pregnant women with an existing diagnosis of periodontitis. During recruitment, participants are allocated to one of four intervention arms using a permutated block randomisation matrix (Fig. 1). The arms are as follows:

1. Semi-skimmed powdered milk fortified with additional sachet of 500 mg calcium and 500 IU vitamin D and early PT (PT during pregnancy)
2. Semi-skimmed powdered milk fortified with additional sachet of 500 mg calcium and 500 IU vitamin D and late onset PT (PT after delivery)
3. Semi-skimmed powdered milk with placebo sachet and early PT
4. Semi-skimmed powdered milk with placebo sachet and late onset PT

The 2 × 2 design allows gathering evidence to feed into an adequately powered RCT to test two different hypotheses, so the four arms will enable estimation of the approximated effect that the specific combinations of the intervention components add to improving concentrations of maternal blood metabolic/inflammation markers and periodontal status.

Baseline data was collected up to second trimester (T0), with follow-up at third trimester (T1) and 6–8 weeks postpartum (T2).

### Questionnaire data

At the three points (T0–T2), the pregnant women with periodontitis diagnosis answer questionnaires about sleep [36], physical activity level [37], frequency of food consumption [38], smoking habits, alcohol consumption, maternal conditions (general questionnaire), pregnancy complications, sunlight exposure, mental health [39], health general [40] and social support [41]. At the baseline, women also provided socioeconomic information. A specific questionnaire on Oral Health Impact Profile (OHIP) [42] was administered at baseline (T0) and 6–8 weeks postpartum (T2) (Fig. 1).

### Biomarkers and measures

Anthropometric (height and weight) and blood pressure are measured in T0–T2. Maternal blood samples were collected in the morning after 12-h overnight fast in the T0, T1 and after delivery (T2) for analysis of biochemical markers. Also 24-h urine is collected at three-time points (T0–T2) for analysis of urinary calcium and creatinine. Prior to the urine collection, all participants are carefully instructed regarding the specific procedures. The blood and urine samples are stored in a freezer with a temperature of − 80 °C until analysis in the Institute of Nutrition Josué de Castro at the Federal University of Rio de Janeiro.

### Recruitment period and setting

The participant recruitment period started in April 2017 and ended in May 2018 with follow-up scheduled until February 2019. The study is conducted in the Municipal Health Centre of Duque de Caxias, Rio de Janeiro/Brazil. This public health centre offers prenatal care for low-risk pregnant women, child health programmes, as well as a clinical laboratory. The population assisted by this centre is of low income and most of them live in the surrounding slums.

Duque de Caxias is a city in the metropolitan region located geographically in the Rio de Janeiro state with approximately 900,000 inhabitants [43]. Duque de Caxias presents a neonatal mortality rate of 12.1 per 1000 live births, 0.7% of maternal deaths investigated and 20.4% of families covered by the family health strategy [44]. These programmes are targeted at families that live under adverse conditions and whose nutritional status is impacted by multiple constraints such as living with some degree of food insecurity (limited access to adequate quantity and quality of food) [45].

The latitude of Duque de Caxias is 22^o^ 47′ S. This area of Brazil is usually sunny all year round. The climate is tropical and summers are hot and humid, while the winters are mild and with more restricted rainfall. The pollution index in this city is high according to the recommendations of the World Health Organization (mean annual concentrations of inhalable particles of diameter less than 10 μm < 20 μg/m^3^ [46], with a mean estimate of 45.9 (SD 21.6) μg/m^3^ between the years 2000–2008 [47].

### Participants and eligibility criteria

The study population includes low-risk adult pregnant women, with periodontitis, attending the prenatal care in the Municipal Health Centre of Duque de Caxias, Rio de Janeiro/Brazil. Inclusion and exclusion criteria are described in Table 2.

**Table 2:** Inclusion and exclusion criteria for the IMPROVE feasibility trial

Inclusion criteria	Exclusion criteria
1. Aged ≥ 18 years at the time of recruitment	1. Positive diagnosis of HIV/AIDS, syphilis, psychosis, diabetes before pregnancy, thyroid disease, or any disorder causing vitamin D hypersensitivity (e.g. sarcoidosis and other lymphomatous disorders)
2. > 20 weeks gestation at first prenatal visit	2. Lactose intolerance, milk allergy, history of renal stones or family history of renal stone and hyperparathyroidism
3. Positive diagnosis of periodontitis (≥ 1 tooth with at least one site with ≥ 4 mm of clinical attachment loss (CAL) and presence of bleeding on probing)	3. Extensive dental cavity (crowns of several teeth destroyed by caries) and loss of tooth structure or use of fixed dental braces
4. Cognitively and physically able to complete an interview and oral examination and willing to participate, including provision of blood samples.	4. Use of antibiotics or any immune-suppressants or medication known to affect vitamin D/calcium metabolism
	5. Consumption of ≥ 4 servings/day of dairy products or taking vitamin D supplements > 400 IU/day

### Recruitment

**Fig. 2:**
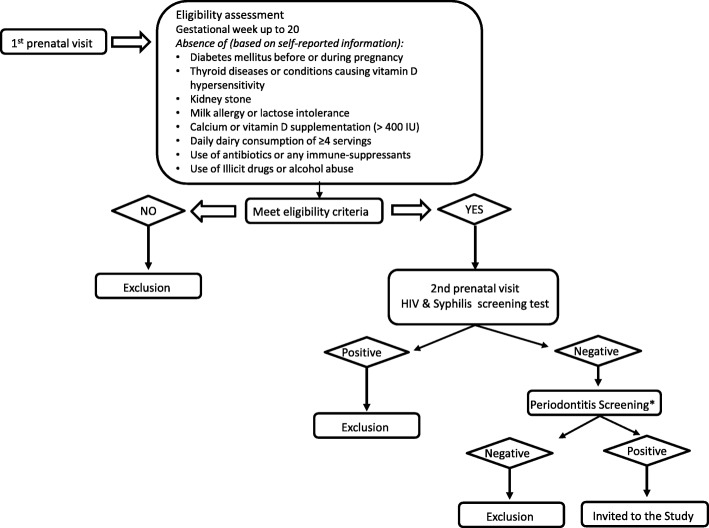
Recruitment of participants *Pregnant women with periodontitis but presenting extensive caries were excluded

In the first prenatal visit a member of the research team introduced pregnant women to the study and invited those initially interested in taking part to answer a preliminary checklist for eligibility, except for the diagnostic of periodontitis. After this, women were screened for syphilis and HIV, as part of the routine pre-natal care. Those who were preliminarily eligible and tested negative for syphilis and HIV were then invited to book a dental examination. Those who screened positive for periodontitis were provided with an informed consent form and included in the study (Fig. 2).

### Randomisation, allocation concealment and blinding

To avoid information bias, regarding influence of interviewer by knowledge of group allocation of participants, randomisation took place after baseline assessment. An online randomisation system with a random mixture of permuted block sizes, stratifying participants by current smoking habit (yes vs. no) was provided and concealed by *Sealed Envelope Ltdtm*.

Due to the nature of the intervention (early vs. late PT), complete blinding of the intervention was not possible. However, dentists and participants were blinded to group allocation regarding milk fortification. Outcome assessment is blinded, and the dentist performing the final periodontal examination is not aware of group allocation regarding both placebo or fortified sachet and PT before or after delivery.

### Interventions

Participants were advised to take two servings of a powdered vitamin D and calcium plus semi-skimmed milk daily during breakfast and afternoon snack or supper to avoid concomitant intake of the prenatal iron supplements routinely prescribed for consumption with hot main meals (e.g. lunch or dinner). Twenty grams (20 g) of powdered semi-skimmed milk (of a commercial brand available in Brazil) is mixed with a sachet (2 g), containing calcium (CAPOLAC 500 mg) [48] and vitamin D_3_ (500 IU), to be reconstituted in 200 mL of potable water for each serving or other preparations (i.e. to consume with kneaded fruits, fruit smoothies, yogurt, porridge, etc.). Sachets with vitamin D/calcium and milk powder were provided monthly to participants in the fortified group during routine prenatal visits.

In the first dental session, a full periodontal examination was repeated to allow comparison with the first examination done during recruitment (screening) and check the agreement between the two dentists. After that, conventional non-surgical PT, consisting of prophylactic dental polishing to remove the sticky bacterial film that forms on the teeth overtime, scaling and root planning, as necessary, was undertaken throughout pregnancy, up to delivery (Fig. 1). The periodontal treatment is carried out at the Municipal Health Centre of Duque de Caxias. The treatment is performed by a qualified and trained dentist who was not involved in the screening procedure and was blind regarding group allocation.

PT started right after the randomisation to ensure early therapy initiation for the relevant treatment arms. The number of treatment sessions depended on the severity of the disease. However, participants were allowed up to five treatment sessions. In each session, participants also received personalised oral hygiene instructions. Standard fluoride toothpaste, without any additional component that could interfere in results, a toothbrush and dental floss were provided to all participants. Monitoring sessions (reassessment of dental conditions) were taken at 2-month interval. If necessary, PT and oral hygiene instructions were repeated.

All participants were advised not to alter their routine physical activity throughout the study and not to consume any supplements other than the ones provided by the clinics. Throughout pregnancy, participants were allowed to consume 400 μg/d folic acid and 60 mg/d ferrous sulphate, as provided during standard prenatal care in Brazil.

### Control group

The control group are the women assigned to the semi-skimmed powdered milk with non-fortified sachet (placebo) with similar colour, flavour, smell and texture as well as before and after dilution than fortified sachet, during pregnancy and late onset PT (after delivery). Late-onset PT starts after postpartum assessment, as part of the routine dental care in the prenatal clinic. This strategy is considered a more acceptable approach in comparison to offering no PT intervention, since the possibility of being assigned to an ‘untreated’ group could limit recruitment and is more ethical.

All participants were informed about the benefits of dairy food intake during pregnancy and given a brochure to take home with general information about healthy diet and oral health hygiene instructions once they entered the study.

### Dental examination

After initial screening, participants underwent a full periodontal examination at baseline (T0) and postpartum (T2). Periodontal examination was performed in full mouth at six sites per tooth (known as mesiobuccal, mid-buccal, distobuccal, mesiolingual, mid-lingual and distolingual), using North Carolina periodontal probes, dental mirror and gauze, but without X-rays. The model chosen is justified because no partial record model presents reliability to replace full mouth examination [49]. The T0 and T2 evaluations take place in the recruitment room, using a simple chair and a forehead flashlight. Maintenance examinations are performed to assess the periodontal status of those who had finished the treatment during pregnancy, and these examinations are done every 2 months. Data collection included gingival bleeding on probing, probing depth, cementoenamel junction-gingival margin distance (CAL) and dental mobility. A tailored form was developed to register the data. Oral examination and treatment procedures were performed by calibrated and trained dentists. The dentists always calibrated their probing force using a scale right before the clinical examination. The recommended probing force was approximately 20 g of pressure [50].

### Process evaluation of qualitative data

Focus group discussions were held prior to trial recruitment to discuss issues regarding recruitment strategy, study design and data collection. In addition, group discussions were held throughout the study (second and third trimester) to assess potential barriers and facilitators to the intervention and data collection.

The process evaluation included qualitative data from focus group and interviews [51, 52].

To perform a content analysis [53], the specific process evaluation frame was developed including two major categories: (1) dietetics abilities to understand the way the women organise daily use of milk sachet with their habitual food and (2) health care and identification of their existing social support network in health care. The evaluation frame was used to generate content associated with two major categories. Six themes were derived from two categories: (1) how to make and consume the food; sharing of the food with other family members; and (2) intolerances social support network and challenges in social life, access to health unit and identification of events related to pain and discomfort. All researchers were trained to fill into a frame of content previously structured with data related to pregnant women’s speeches associated to the six themes described above. Initially, one pilot focus group was performed with five women of similar socioeconomic conditions to those attending the health clinic where the present study is taking place. After some adjustments in the content of questions, the data was coded into themes and classified into three levels to identify favourable factors and health events: favourable, not favourable and neutral.

Second focus group regarding the culinary knowledge, health care practices including oral health and network social support at Duque de Caxias territory was performed prior to recruitment with 13 pregnant women attending the health centre. Thus, in the present study, we evaluated some of the barriers and enablers to participation, recruitment and intervention delivery, as well as the difficulties to go to the health unit.

Group discussions were held during the study after recruitment to assess potential barriers and facilitators to the intervention delivery and data collection. These group discussions provided qualitative data about potential acceptability. Additionally, monthly individual meetings were organised in the health centre with participants throughout the study to complement and update the qualitative data collection. Sentences and phases were registered and added into the frame, and frequency of occurrences, the patterns and the sequence of data are analysed. These data were then used to assist follow-up management and improve compliance with the intervention (e.g. suggestions on how to overcome reported barriers regarding milk consumption or monotonous diet). At the end of each participant’s involvement in the study, they were asked to complete an anonymous evaluation questionnaire to gain insights about women’s participation experience, acceptability and compliance to the study protocol.

### Adherence

Adherence were routinely assessed counting the unused sachets monthly, i.e. number of returned sachet by pregnant women in their monthly visit. SMS reminders to consume the sachets and milk were sent (once a week) to participants to help prevent attrition. A surplus of plain milk powder was provided to all women with young children to ensure dose fidelity. Acceptance of the intervention was evaluated via a questionnaire comprising questions about quality of taste (both milk powder with fortified and placebo sachet), possible difference in taste between those provided by the study and the commercial ones that they had tried. The amount of sachet consumed and suggested time of consumption, as well as the continuation of the program were evaluated.

Moreover, in the focus group, the issues raised regarding barriers to comply with the intervention protocol were addressed (e.g. new recipes are provided to women who complain about monotonous diet; possibility of home delivery of the powdered milk and sachet, etc.).

A social media group in a free mobile app was created to promote participant engagement and peer interaction. This strategy may reduce the attrition rate and improve the commitment with the project.

### Outcome measures

#### Primary outcome measures

1. Feasibility (acceptability of study design, recruitment strategy, random allocation and data collection procedures).

Feasibility will be evaluated using mixed methods to explore intervention delivery, participants’ acceptability, challenges and issues faced during the study and recommended changes to the study design. Moreover, the number of protocol deviation; an estimate of the cost of carrying out the study; time required for recruitment, data collection, and analysis will be evaluated.

2) Recruitment rate

The total number of participants recruited into the study, number of participants recruited per month, number of invited women, and number of excluded participants before and after the dental screening and reasons for exclusion.

3) Adherence

Percentage of sachet consumed during the study per group (based on the number of returned sachet by pregnant women in their monthly visit); average number of follow-up visits, percentage of participants who provide full data at baseline throughout pregnancy and up to 6–8 weeks postpartum.

4) Attrition rate

Number of dropouts in each study arm and reasons for dropout.

#### Secondary outcome measures

1. Changes in mean and SD of percentage of sites with bleeding on probing (BOP) and CAL between postpartum and baseline
2. Changes in maternal blood levels of biomarkers of inflammation (CRP, IL-6 and MMP-9)
3. Changes in maternal blood levels of 25(OH) D, glucose, insulin, calcium, parathyroid hormone and lipids (HDL, LDL and triglycerides)
4. Neonatal blood levels of 25(OH) D and calcium

### Sample size

This is a feasibility study, and thus the final sample size will be determined by the number of participants that are feasible to recruit within the timescale and budget of the project. For feasibility studies, a minimum of 12 subjects per group is proposed by Julious [54], and a total sample size of 70 subjects is proposed by Teare et al. [55] for a continuous outcome. Since we are planning a 2 × 2 factorial RCT, a sample size of 120 (30 per group), would be enough to detect a realistic event and allowing for 58% dropout rates (considering a sample size of 70), with sufficient precision and minimal bias for the estimates, as well as the existence of an additive interaction between the two interventions.

### Patient and public involvement

Initially, we held meetings with directors and health professionals at the centre to get details on the prenatal care routine and logistics, socioeconomic and demographic profile of service users, numbers of pregnant women attending per month, and whether the centre offered free dental treatment and nutritional counselling for pregnant women. Subsequently, managers and health professionals at the centre were consulted on how to implement the project with minimal interference in their routine practice. Also, informal consultations with female heath care users (*n* = 4) and pregnant women (*n* = 15) attending the centre were performed to know their opinion about the study, if they considered it interesting for the population, if they had any suggestions on how the project could be implemented, how researchers could invite pregnant women to participate, how the pregnant women would adhere to the proposed protocol, if they would consume the milk and if they had any suggestion on how the team could help in the maintenance of the consumption until the end of the gestation. The views and suggestion of health care professionals and service users were taken into consideration during the implementation of the project.

### Statistical analysis

Baseline characteristics of participants will be summarised using descriptive statistics (i.e. means and standard deviations for normally distributed continuous variables, medians and ranges for skewed continuous variables, frequencies and proportions for categorical/binary variables), for all four intervention arms and total sample. All analyses will be according to the intention-to-treat approach, including all participants, regardless of adherence to the study protocol. To determine the effects of the intervention, time and group-time interaction on outcomes (inflammatory biomarker concentrations and periodontitis status), one-way repeated measures ANOVA will be used. Intervention will be regarded as a between-subject factor and time with three-time points (T0, T1 and T2) as a within-subject factor. Mean change of biomarker concentrations, number of sites with bleeding on probing and clinical attachment level (periodontal status) will be compared between the four treatment groups using ANCOVA, adjusted for their baseline values. A *P* value < 0.05 will be considered statiscally significant.

### Data management

Questionnaires (quantitative data) are performed directly using RedCap software (Research Electronic Data Capture), with features that minimises data entry errors and facilitates conversion to other statistical software for data analysis. Questionnaires were designed specifically so that data entry in RedCap was made as accurate as possible.

Questionnaires are peer reviewed before data entry to search for missing data, erroneous values and inconsistency*.* The identified problems are being solved (data cleaning) using Stata 12 software (Stata Corp 12, TX, USA). Quantitative data are being held in Stata data file (.dta), which has the capacity to store hundreds of variables. Stata software is widely used in research and files can be easily converted into other formats via Stata transfer software. Qualitative interviews are being audio recorded (with full participant consent) and stored in MP3 files. Transcripts are analysed by thematic content analysis and Nvivo software for supporting qualitative analysis is used.

All laboratory specimens, evaluation forms, reports, and other records are identified in a manner designed to maintain participant confidentiality. All records are kept in secure cabinets with limited access. Clinical information cannot be released without the written permission of the participant, except as necessary for monitoring and auditing by the sponsor and regularity authorities. The investigator and the study site staff involved in this study are not allowed to disclose or use for any purpose other than performance of the study.

Computers used to collate the data have limited access measures via user names and passwords. Published results will not contain any personal data that could allow the identification of individual participants. All confidential data (name, address, etc.) are archived separately from research data. Data are housed on a secure server protected by university firewalls and are subject to backups.

### Conditions for discontinuation of participation in this clinical trial

1. If a pregnant woman withdraws consent for participation in the clinical trial
2. If a participant is diagnosed after recruitment with serious kidney disease, or other disease (i.e. lactose intolerance, milk protein intolerance), which vitamin D/calcium supplementation or milk consumption are not recommended

### Protocol amendments

The Ethics Committee will be informed if any amendments of the protocol are planned, and these amendments will only be implemented after approval.

### Follow-up of adverse events

No risky procedure will be employed in this study, and the proposed interventions are considered safe for both the mother and the foetus. However, calcium supplementation in pregnancy may cause constipation. To circumvent this potential side effect, women are being advised to drink enough fluids throughout the study.

Studies have shown that foetal excess of vitamin D metabolites are unlikely to occur when maternal concentrations are within a normal range [56]. Therefore, offering a dose of 1000 IU vitamin D to women during pregnancy would be unlikely to cause any toxicity effect. Recent studies have not identified adverse effects in pregnant women and/or their children with the ingestion of vitamin D supplementation above 1000 IU daily administered during pregnancy [57–59]. Furthermore, the maximum tolerable upper intake level of vitamin D in pregnant women is 4000 IU/day. This quantity of vitamin D a day to pregnant women not only was associated with any toxicity but that it also was associated with better birth outcomes, i.e. improvement in gestational age at birth [34].

In a Cochrane review, the authors concluded that vitamin D supplementation, alone or in combination with calcium and micronutrients, and the low side effect profile of vitamin D is a cost-effective measure and favourable for use in under-resourced settings [60].

Regarding PT, it has been speculated whether periodontal mechanical manipulation would cause bacteraemia, which itself could initiate the pathway leading to adverse pregnancy outcomes [24]. However, a large RCT showed that rates of adverse outcomes did not differ significantly between women who received treatments and those who did not require treatment. Use of topical or local anaesthetics during root planning was not associated with an increased risk of experiencing adverse outcomes. The authors concluded that essential dental treatment for moderate-to-severe caries or fractured or abscessed teeth and periodontal treatment are safe for pregnant women [61].

Venipuncture in newborns is impractical and collection of cord blood imposes a logistic burden to the hospital staff. Alternatively, drops of neonatal capillary blood will be collected via heel prick for measurement of calcium and 25(OH) D concentrations at the routine neonatal check-up. This test is harmless, with minimal discomfort. The amount of blood taken is too small to be likely to have any adverse effects.

Despite the minimal risks anticipated for this study, any adverse event or serious adverse event will be described according to its relatedness with the interventions, for both the total sample and by treatment arms, in the final study report.

### Dissemination plan and impact

The main results of this study will be presented to the director, participants, prenatal nurses and physician of the Municipal Health Centre of Duque de Caxias, Rio de Janeiro/Brazil, through a workshop given by the researchers. The results will also be presented at national and international conferences and published in scientific journals in accordance with the CONSORT statement [62] and its extensions relating to non-pharmacological studies [63, 64] and TIDieR (template for intervention description and replication) [65] guidelines for intervention description and replication. Additionally, the results of this feasibility clinical trial could inform the study design of a full RCT in pregnant women with periodontitis.

## Discussion

Periodontitis and vitamin D deficiency among pregnant women have been highly associated with adverse maternal-foetal outcomes including increased risk of delivering preterm and low-birth-weight newborns [13–15]. Despite the evidence on negative pregnancy outcomes, the prevalence of periodontitis and maternal vitamin D deficiency is still high in both developing countries and developed countries.

There is no consensus between studies that evaluate the association between PT alone during pregnancy with improvements of periodontitis [20–23]. However, some authors have observed that the occurrence of periodontitis is less frequent among adults consuming calcium within recommendations and higher quantities of vitamin D [28, 29] and that vitamin D and calcium co-supplementation seems to improve metabolic profile of pregnant women [27]. Moreover, there is limited but suggestive evidence of positive association between milk consumption and foetal growth and infant birthweight in healthy Western populations [66].

Due to the fact that oral health and mineral/vitamin supplementation are still overlooked in the prenatal assistance in Brazilian and there is scarcity of clinical trials addressing both oral health and calcium and vitamin D deficiency, the present study was designed for assessing the feasibility of a RCT on acceptability of a multi-component intervention combining conventional periodontal treatment and milk fortified with calcium-vitamin D intake for improving periodontal conditions and maternal metabolic and inflammation status, among Brazilian low-income pregnant women with periodontitis.

Policy makers, administrators and clinicians need to take the necessary steps to eliminate this easily preventable threat to mothers and infants. Thus, we hope that this relatively low-cost and safe multicomponent intervention can help reduce inflammation and improve maternal periodontal.

## Abbreviations

BOP: Bleeding on probing
CAL: Clinical attachment loss
CRP: C-reactive protein
IL-6: Interleukin-6
IMPROVE: This feasibility randomised controlled trial
MMP-9: Matrix metalloproteinase 9
OHIP: Oral Health Impact Profile
PT: Periodontal therapy
RCT: Randomised controlled trials
TIDieR: Template for intervention description and replication health and metabolic profile and consequently prevent negative gestational outcomes
