# Correction to: Calcium and vitamin D supplementation and/or periodontal therapy in the treatment of periodontitis among Brazilian pregnant women: protocol of a feasibility randomised controlled trial (the IMPROVE trial)

**DOI:** 10.1186/s40814-020-00727-6

**Published:** 2020-11-27

**Authors:** Paula Guedes Cocate, Gilberto Kac, Berit Lilienthal Hieitmann, Paulo Nadanovsky, Maria Cláudia da Veiga Soares Carvalho, Camila Benaim, Michael Maia Schlüssel, Maria Beatriz Trindade de Castro, Nadya Helena Alves-Santos, Amanda Farnum Baptista, Michael F. Holick, Rana R. Mokhtar, Alessandra Raymundo Bomfim, Amanda Rodrigues Amorim Adegboye

**Affiliations:** 1 Department of Biosciences and Physical Activity, School of Physical Education and Sports, Federal University of Rio de Janeiro, Rio de Janeiro, Brazil; 2 Nutritional Epidemiology Observatory, Department of Social and Applied Nutrition, Institute of Nutrition Josué de Castro, Federal University of Rio de Janeiro, Rio de Janeiro, Brazil; 3 Research Unit for Dietary Studies at the Parker Institute, Bispebjerg and Frederiksberg Hospital, The Capital Region, Denmark and Section for General Medicine, Institute of Public Health, Copenhagen, Denmark; 4 Institute of Social Medicine, State University of Rio de Janeiro, Rio de Janeiro, Brazil; 5 Department of Social and Applied Nutrition, Institute of Nutrition Josué de Castro, Federal University of Rio de Janeiro, Rio de Janeiro, Brazil; 6 The EQUATOR Network – UK Centre, Centre for Statistics in Medicine, Nuffield Department of Orthopaedics, Rheumatology and Musculoskeletal Sciences, University of Oxford, Old Road, Oxford, OX3 7LD UK; 7 Section of Endocrinology, Diabetes & Nutrition, Department of Medicine Boston University School of Medicine (BUSM), Boston, Massachusetts United States; 8 School of Human Sciences, Faculty of Education, Health and Human Sciences, University of Greenwich, London, UK

**Correction to: Pilot Feasibility Stud 5, 38 (2019)**

**https://doi.org/10.1186/s40814-019-0417-6**

Following publication of the original article [1], the authors reported errors that need to be corrected. The errors consist of text omission as followsr

The last paragraph under **Study design and setting** section reads ‘Baseline data is collected up to the second trimester after checking for participants eligibility to the study (including dental screening for periodontitis) and prior to randomisation to intervention arms (T0); with follow up at the third trimester (T1; during the intervention) and 6-8 weeks postpartum (T2; after the intervention).’

Item 4 (Attrition rate) under **Outcome Measures** section the text reads ‘To evaluate the reliability and completeness of outcome a feasibility threshold of 70-75% for recruitment will be considered as adequate. Furthermore, an adherence rate of 70% and a loss to follow-up between 20-30% will be considered as adequate. Adequate acceptance of the study design and intervention will be based on qualitative data on barriers and facilitators to the intervention and taste test, the number of sachets consumed and feedback from the follow-up phone calls.’

In the **Statistical analysis** section, an additional paragraph is included. ‘It is important to highlight that the present study is a feasibility trial. Thus, the priority will be performance of descriptive analysis and estimation of sample size for the main study, therefore the statistical analysis related to results from hypothesis should be interpreted with caution [56] and viewed as entirely exploratory’.

In the **Discussion** section, a final paragraph was included.

‘This study has several potential limitations. Since aggressive periodontitis has a relatively low prevalence based on finding from two systematic reviews [68, 69], it is unlikely that women with this condition will be recruited to the study. However, this chance cannot be completely ruled out as the diagnosis of such condition in resource-limited settings (such as public health clinics in low-income areas in Rio de Janeiro, Brazil) might be complex. Although having dental X-rays during pregnancy might be considered safe [70, 71] we did not have ethical approval to conduct such procedures. Similar to previous studies on periodontitis status among pregnant women, an X-ray was not taken to help confirm the diagnosis [72, 73, 74]. ‘.

**Funding:** The funders did not contribute to the study design, data collection and management. They will not interfere with data analysis and interpretations of findings.

**Updated Affiliations**

Paula Guedes Cocate ^1^ Department of Biosciences and Physical Activity, School of Physical Education and Sports, Federal University of Rio de Janeiro

Michael Maia Schlüssel ^**6**^ The EQUATOR Network – UK Centre, Centre for Statistics in Medicine, Nuffield Department of Orthopaedics, Rheumatology and Musculoskeletal Sciences, University of Oxford, Old Road, OX3 7LD Oxford, UK

Rana R. Mokhtar ^7^ Section of Endocrinology, Diabetes & Nutrition, Department of Medicine Boston University School of Medicine (BUSM), Boston, Massachusetts, United States.

Amanda Rodrigues Amorim Adegboye ^8^ School of Human Sciences, Faculty of Education, Health and Human Sciences, University of Greenwich, London, UK

The **reference list** was updated from reference 56 onwards.

56. Arain M, Campbell MJ, Cooper CL, Lancaster GA. “What is a pilot or feasibility study? A review of current practice and editorial policy.” BMC Medical Research Methodology. 2010; 10:67. 10.1186/1471-2288-10-67

57. Dror DK, Allen LH. Vitamin D inadequacy in pregnancy: biology, outcomes, and interventions. *Nutr Rev* 2010;68(8):465–77.

58. Rodda CP, Benson JE, Vincent AJ, et al. Maternal vitamin D supplementation during pregnancy prevents vitamin D deficiency in the newborn: an open-label randomized controlled trial. *Clin Endocrinol (Oxf)* 2015;83(3):363–8.

59. Dawodu A, Saadi HF, Bekdache G, et al. Randomized controlled trial (RCT) of vitamin D supplementation in pregnancy in a population with endemic vitamin D deficiency. *J Clin Endocrinol Metab* 2013;98(6):2337–46.

60. Hollis BW, Johnson D, Hulsey TC, et al. Vitamin D supplementation during pregnancy: double-blind, randomized clinical trial of safety and effectiveness. *J Bone Miner Res* 2011;26(10):2341–57.

61. De-Regil LM, Palacios C, Ansary A, et al. Vitamin D supplementation for women during pregnancy. *Cochrane Database Syst Rev* 2012(2):CD008873.

62. Michalowicz BS, DiAngelis AJ, Novak MJ, et al. Examining the safety of dental treatment in pregnant women. *J Am Dent Assoc*2008;139(6):685–95.

63. Schulz KF, Altman DG, Moher D. Group C. CONSORT 2010 statement: updated guidelines for reporting parallel group randomized trials. *Obstet Gynecol* 2010;115(5):1063–70.

64. Boutron I, Moher D, Altman DG, et al. Group C. Extending the CONSORT statement to randomized trials of nonpharmacologic treatment: explanation and elaboration. *Ann Intern Med* 2008;148(4):295–309.

65. Zwarenstein M, Treweek S, Gagnier JJ, et al. Improving the reporting of pragmatic trials: an extension of the CONSORT statement. *BMJ* 2008;337:a2390.

66. Hoffmann TC, Glasziou PP, Boutron I, et al. Better reporting of interventions: template for intervention description and replication (TIDieR) checklist and guide. *BMJ* 2014;348:g1687.

67. Brantsæter AL, Olafsdottir AS, Forsum E, Olsen SF, Thorsdottir I. Does milk and dairy consumption during pregnancy influence fetalgrowth and infant birthweight? Asystematic literature review. *FoodNutr Res*2012;56.

68. Susin C, Haas AN, Albandar JM. Epidemiology and demographics of aggressive periodontitis. Periodontol 2000. 2014; 65: 27–45. doi: 10.1111/prd.12019.

69. Demmer RT, Papapanou PN. Epidemiologic patterns of chronic and aggressive periodontitis. Periodontol 2000. 2010; 53: 28–44. doi:10.1111/j.1600-0757.2009.00326.x.

70. American Dental Association, Council on Scientific Affairs, U.S. Food and Drug Administration. 2012. Dental Radiographic Examinations: Recommendations for Patient Selection and Limiting Radiation Exposure (rev.). Chicago, IL: American Dental Association. http://www.ada.org/~/media/ ADA/Member%20Center/FIles/Dental_Radiographic_Examinations_2012.aspx.

71. ACOG Committee Opinion. Guidelines for Diagnostic Imaging During Pregnancy and Lactation. The American College of Obstetrician and Gynecologists. 2017; 130(4):e210-e216.

72. Jaiman G, Nayak PA, Sharma S, Nagpal K. Maternal periodontal disease and preeclampsia in Jaipur population. J Indian Soc Periodontol. 2018 Jan-Feb;22(1):50–54. doi: 10.4103/jisp.jisp_363_15.

73. Machado V, Mesquita MF, Bernardo MA, Casal E, Proença L, Mendes JJ. IL-6 and TNF-α salivary levels according to the periodontal status in Portuguese pregnant women. PeerJ. 2018 May 4;6:e4710. doi: 10.7717/peerj.4710. eCollection 2018.

74. Trindade, Soraya Castro, Barreto, Juliana Albuquerque Reis, Barreto Neto, Laerte Oliveira, Passos-Soares, Johelle de Santana, Vianna, Maria Isabel Pereira, Azevedo, Antônio Cesar Oliveira, Genovese, Walter João, Barreto, Maurício Lima, Cruz, Simone Seixas da, & Gomes Filho, Isaac Suzart. (2018). Oral health status of pregnant and puerperal women in the municipality of Feira de Santana, at three different times between 2005 and 2015. Epidemiologia e Serviços de Saúde, 27(3), e2017273. Epub September 21, 2018.10.5123/s1679-49742018000300009

## References

[1] Cocate PG, Kac G, Heitmann BL (2019). Calcium and vitamin D supplementation and/or periodontal therapy in the treatment of periodontitis among Brazilian pregnant women: protocol of a feasibility randomised controlled trial (the IMPROVE trial). Pilot Feasibility Stud.

